# A qualitative study of culturally embedded factors in complementary and alternative medicine use

**DOI:** 10.1186/s12906-018-2093-0

**Published:** 2018-01-22

**Authors:** Szilvia Zörgő, György Purebl, Ágnes Zana

**Affiliations:** Semmelweis University, Institute of Behavioural Sciences, 4 Nagyvárad tér, Budapest, 1089 Hungary

**Keywords:** Qualitative, Medical anthropology, Complementary and alternative medicine, Therapy choice, Traditional Chinese Medicine

## Abstract

**Background:**

Within the intercultural milieu of medical pluralism, a nexus of worldviews espousing distinct explanatory models of illness, our research aims at exploring factors leading to complementary and alternative medicine (CAM) use with special attention to their cultural context.

**Methods:**

The results are based on medical anthropological fieldwork (participant observation and in-depth interviews) spanning a period from January 2015 to May 2017 at four clinics of Traditional Chinese Medicine in Budapest, Hungary. Participant observation involved 105 patients (males *N* = 42); in-depth interviews were conducted with patients (*N* = 9) and practitioners (*N* = 9). The interviews were coded with Interpretative Phenomenological Analysis; all information was aggregated employing Atlas.ti software.

**Results:**

In order to avoid the dichotomization of “push and pull factors,” results obtained from the fieldwork and interviews were structured along milestones of the patient journey. These points of reference include orientation among sources of information, biomedical diagnosis, patient expectations and the physician-patient relationship, the biomedical treatment trajectory and reasons for non-adherence, philosophical congruence, and alternate routes of entry into the world of CAM. All discussed points which are a departure from the strictly western therapy, entail an underlying socio-cultural disposition and must be scrutinized in this context.

**Conclusions:**

The influence of one’s culturally determined explanatory model is ubiquitous from the onset of the patient journey and exhibits a reciprocal relationship with subjective experience. Firsthand experience (or that of the Other) signifies the most reliable source of information in matters of illness and choice of therapy. Furthermore, the theme of (building and losing) trust is present throughout the patient journey, a determining factor in patient decision-making and dispositions toward both CAM and biomedicine.

## Background

Although a myriad of surveys support that complementary and alternative medicine (CAM) is becoming increasingly popular among European and North American populations [1], there are ambiguities in defining CAM as well as statistical discrepancies due to varying research design. CAM can be described as “diverse therapies that are not commonly available through conventional medicine outlets nor commonly taught in conventional medical schools” [2], yet what is commonly accepted by the professional sector of the healthcare system differs not only among countries but among hospitals within one country as well. Adding to the complexity of the matter is that criteria for treatments (eventually) comprising a part of conventional medicine are in close connection with whether they are evidence-based or not – a determinant that is difficult to reach consensus on, i.e. what precisely constitutes “scientific evidence.”

Statistical analysis on an international scale concerning CAM use may be misleading because these results cannot be interpreted without their cultural context and with special attention to research design. For example, one study shows 73.5% of cancer patients in Ghana employ CAM [3], but 70% of the Ghana population cannot afford Western medicine to begin with, furthermore cancer patients are often diagnosed too late to receive biomedical treatment [3]. HIV/AIDS patients were involved in a study regarding CAM use in Malaysia, which yielded results of 78.2% [4], but the sample was restricted to only one hospital. Research design also involves limiting the CAM under scrutiny, which in the case of cancer patients’ CAM use in America [5] (34% CAM use, 212 subjects, 1st phase cancer) involved only examining “biologically-based” CAM modalities and disregarding all others. Research design may also involve limiting the study to a certain illness, as in a Swedish exploration of CAM use solely among lung cancer patients (53% CAM use) [6]. Thus, statistics are highly dependent on cultural context, CAM under scrutiny, and research design and it is difficult to confidently navigate within the skein of CAM use-related information.

Although, there are general surveys, which do provide a more accurate picture of society-level CAM use, such as the 2002, 2007, 2012 NHIS survey of the US population [7] that found 36%–42% of adults employed a CAM modality, showing an increase from previous studies [8–10]. A 2012 systematic literature review of CAM prevalence among adults in the EU found results as high as 86% [11], yet a tendency in which studies concur is that CAM use is on the rise [2, 12–14] and the CAM market is amid intense diversification, which poses a significant challenge in CAM categorization. This is not only a problem of what conventional medicine accepts as a part of the professional sector, but also a question of an appropriate typology for those modalities that are on the periphery of or out of bounds relative to biomedicine. Scientific discourse on the topic usually adheres to the categorization of the US National Center for Complementary and Integrative Health (NCCIH), but this typology leaves a significant amount of interpretative space open to subjective judgment.

The NCCIH categorization comprises five major domains: alternative medical systems, mind-body interventions, biologically-based treatments, manipulative and body-based methods, and energy therapies. In a survey asking subjects to categorize their CAM use, one could easily classify a modality, such as Traditional Chinese Medicine (TCM) as an alternative medical system, just as well as a mind-body intervention. The NCCIH typology itself states TCM is an alternative medical system, while classifying acupuncture, a main treatment type employed in TCM, as a mind-body intervention. This has a significant effect on data analysis, as does the fact that naturopathy is appraised as an alternative medical system when in fact it signifies many different modalities, not all embedded in a medical subculture. Furthermore, questions arise regarding how to interpret a modality as belonging to a “medical system,” what connotes a medical system at all. As a final point, if one regularly takes advantage of such family or home remedies as chamomile tea or other herbs, would this be classified as phytotherapy or biologically-based CAM therapy use? Therefore, many challenges arise not only in research design and data interpretation, but also in defining what CAM is (where the limits of biomedicine reside) and how to develop a functioning typology. These questions transcend the scope of this paper, nevertheless they underlie quantitative results concerning CAM use.

The increasing use of CAM and its proliferation on the healthcare market signifies an increasing demand for such modalities among the general population, and simultaneously suggests that these modalities (whether they be alternative medical systems or not) have a significant effect on health-related concepts and decision-making. In terms of a pluralistic healthcare market, biomedicine is also “only” one economic actor, albeit the most dominant; the complexity of biomedicine’s position in this market is exemplified by the intricacies of patients’ choice of therapy. An individual’s decisions are always in dialogue with the greater societal structure and values [15] and must be interpreted in their context. The following aims to provide a global and societal context for individual decisions concerning therapy choice, as listing factors leading to CAM use are not sufficient in themselves, these factors are embedded in a cultural milieu.

Many authors [9, 16–21] have dichotomized factors leading to CAM use, grouping these reasons into “push factors” (repelling the patient from biomedicine) or “pull factors” (attracting patients to CAM). As we shed light onto individual cases, we may encounter the complexity of the decision making process, finding that “push and pull factors” are frequently so intertwined that it may not make analytical sense to distinguish such categories. Stratton argues that CAM fills a market niche, it is strong where biomedicine is lacking: mostly within the arena of coping with and giving meaning to pain and suffering ([15], cf.: [22]). Yet the latter interpretation may not be sufficient in explaining the multitude of reasons why a patient would opt for CAM treatment, neither is conceptualizing CAM use as receiving impetus solely from biomedical deficits.

Connor analyzes CAM use focused on the lay concept of the “natural” (CAM remedies are believed to be harmless and preferred because of this), its inverse, the concept of “toxic” (pharmaceuticals are rejected because they are believed to be harmful), and a type of personal or social resistance to “the hazards of modernity” as the author states [23]. The benefits of such a psycho-social approach to understanding CAM use are immense, as is Kelner and Wellman’s initiative highlighting the importance of the increasing number of “smart consumers” in Western society as an aspect of multifactorial CAM use [9]. Congruently with the latter approach, it is well-worth scrutinizing CAM within the framework of the supply and demand of the healthcare market, yet taking one further step into the socio-cultural domain.

Competition in the healthcare market is not restricted to contending therapeutic products and procedures, but also involves the explanatory models (concepts of world, man, and illness) underlying various medical systems. Most available therapies are culturally embedded and from the vantage point of the patient and practitioner employing them, or the researcher examining associated phenomena, these therapies cannot be isolated from their cultural context. Hence there is also a “cultural market” that, according to Molnár [24], consists of competing and oftentimes conflicting ideologies in which one may frequently encounter contradictory claims and, lacking previous sources of orientation (societal institutions of authority, such as the church), one is left to their own devices in constructing their subjective worldview [25]. Contradictory claims also prevail in health-related topics, as Van Wolputte writes: “The human body emerges as the meeting ground of both hegemony and counterhegemonic practices, power and defiance, authority and subversion” [26]. Consequently, competition among various medical systems also entails an interaction of their cultural phenomena; the stakes of the competition is professional prestige, which signifies more than just popularizing a certain type of medicine, it also establishes the dominance of the worldview it is embedded in and defines the relative position of other cultural/medical systems vis-à-vis societal values.

The abundance of accessible ideologies and practices is a co-phenomenon of globalization, postmodernism and of the decreasing hegemony of societal institutions. One result of this milieu is “cultural creolization”; a social and individual process denoting the integration of concepts and traditions from varying cultures. On the individual level, it can signify an identity-building mechanism: “We all are Creoles of sorts: hybrid, divided, polyphonic, and parodic – a pastiche of our Selves. This contemporary body-self is fragmentary, often incoherent and inconsistent, precisely because it arises from contradictory and paradoxical experiences, social tensions, and conflicts” [26]. Perhaps an equivalency can be drawn with what Ray calls “cultural creatives,” who constitute around 25% of the American adult population [15], and share such values as ecological sustainability, a preference for the exotic and foreign, social optimism, spirituality, and mind-body unity. According to Stratton, cultural creatives represent “the core market” for CAM ([15], cf.: “glocality” [25]).

Thus, as suggested above, CAM use is a complex phenomenon requiring an analysis based on a multitude of perspectives, moving beyond the approach of “push and pull”. Proposed hereafter is a panoply of CAM use factors based on the trajectory of various patient journeys with special attention to psycho-social and cultural context.

## Methods

This study is part of a qualitative research project exploring factors leading to CAM use within the greater context of patient explanatory models (EM) and patient journeys (PJ); this systemic perspective is employed in order to gain more thorough insight into CAM use. Research is limited to Traditional Chinese Medicine (TCM), as the most widespread modality of CAM in Hungary; such clinics provide an anthropological field that can be scrutinized in anticipation of arriving to transferable findings regarding CAM.

The research is founded on cultural anthropological fieldwork, i.e. participant observation and in-depth interviews, conducted between January 2015 to May 2017 at four TCM clinics located in Budapest, Hungary, alongside Hungarian and Chinese practitioners. The participant observation entailed observing the admittance of new patients/first consultations, as well as participating in everyday work performed at the clinics during which unstructured interviews were conducted with patients and practitioners. The attained information was continually recorded in a field journal.

In-depth interviews were conducted with patients undergoing regular treatment and with practitioners at the sites of fieldwork and at other TCM clinics. The semi-structured patient interviews were conducted according to the following thematic blocks: world (attitudes regarding life in general, ontological concepts, religious or faith-based concepts, etc.), man (constituents of man and concepts regarding their interplay), illness (illness definition, typology, concepts regarding present illness), health and healing (definition and related concepts), patient journey (from illness onset to present) and therapy choice (why the patient chose TCM, how they came into contact with it, their evaluation of therapeutic efficacy, etc.). The semi-structured practitioner interviews included some of the above blocks (world, man, illness, health and healing) and also involved a block on their motivations to become a TCM practitioner. The thematic blocks were comprised of subtopics (see above) and probes, the blocks did not have any specific order, but all interviews conducted delved into every block. The interviews were recorded, transcribed verbatim, and coded with Interpretative Phenomenological Analysis [27]. Patient interviewees participated in a longitudinal study whereby follow-up unstructured interviews were conducted at irregular intervals to inquire about changes in condition, illness perception, subjective evaluation of treatment efficacy and other CAM therapies employed.

The anthropological fieldwork (participant observation; unstructured, in-depth and follow-up interviews) was conducted by the same author (Author 1); all the information was continually pooled into a master Hermeneutic Unit within Atlas.ti. In accordance with the systemic perspective on CAM use, the topics listed below signify areas of focus throughout the research and comprise the main structure of the coding system (master codes):

### Explanatory model codes

EM 1: Concepts of world (personal ideology, religious and/or spiritual concepts, relation to societal norms), EM 2: Concepts of man (constituents of man and their interplay), EM 3: Concepts of illness and health (definition of illness and health; illness perception).

### Patient journey codes

PJ 1: Timing of choosing TCM within the context of the patient journey, PJ 2: Immediate reasons behind TCM use, PJ 3: Ongoing or discontinued relation to biomedicine, PJ 4: Relation to other CAM modalities.

**Fig. 1:**
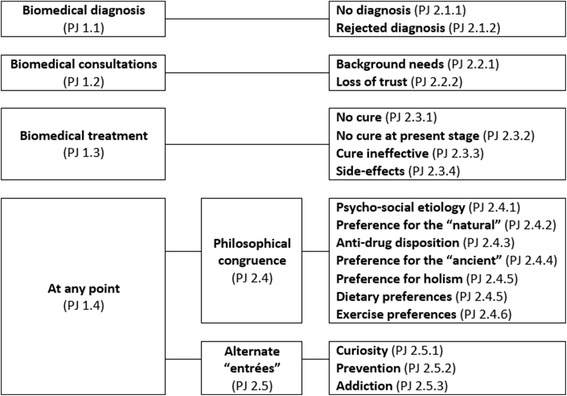
Integrated code tree of PJ 1 and PJ 2 codes

The master codes are based on our preconception that therapy choice is in interaction with the patient’s explanatory model and that the patient journey will reflect decisions made in accordance with the explanatory model. All in-depth patient interviews addressed the above topics, unstructured interviews addressed a sub-set of these, depending on the social circumstance (i.e. available time, level of intimacy, etc.) defined by the field. Findings presented in the article concern codes PJ 1 and PJ 2, while employing narratives to contextualize and illustrate the results. Based on preliminary fieldwork, Author 1 performed the primary coding of the field journal through inductive analysis [28] and developed the code structure (Fig. 1).

In accordance with comparative techniques [29], the code structure was treated dynamically for three months of fieldwork, while the authors performed cross-checking for rivalling code variations. During month 4 of fieldwork, the inductively created code structure was finalized and master codes PJ 1 and PJ 2 were given a separate Hermeneutic Unit within Atlas.ti. At two thirds of the fieldwork (month 20), an uncoded version of the PJ 1–2 Hermeneutic Unit was analyzed by Author 2 and Author 3, working separately, but employing the same code structure for deductive coding (PJ 1.1–1.4 and PJ 2.1–2.5). Following aggregated analysis, a Hungarian version of the findings was shared with 4 interviewees and subsequent to respondent validation, the English article was drafted. Information quoted directly from the field journal or interviews will be shown in italics.

### Sociocultural context of research

Legislation in Hungary divides CAM modalities into two categories. Practitioners of certain modalities constituting one group are required to obtain a medical degree, as in the case of TCM, while there is no such restriction concerning the other group of CAM (cf: [30]). Making a distinction amongst various CAM modalities both exhibits and influences the cultural preferences of the majority of society, as well as expresses the degree of scientific approval and the general political interests concerning particular medical systems/treatments. As evidence-based medicine maintains its dominant role in the healthcare system and in the cultural dispositions of patients, if practitioners of a CAM modality are required to be medical doctors, it raises the prestige of that modality. Also, the political value of a certain CAM modality has a strong effect on its accessibility and stature, as a medical system may signify a marketable cultural commodity, an instrument of professional and cultural collaboration. TCM currently denotes such a cultural commodity in Hungary illustrated in an initiative to establish a Central and Eastern European TCM Center in Budapest that would facilitate educational, research and therapeutic activities, as well as stimulate political and economic cooperation among China and countries of the Central-eastern European region, mediated by Hungary.

Two of the four locations of the anthropological fieldwork discussed below are TCM clinics functioning as a “social hub” of information exchange and cultural creolization. Information exchange is reified by the proxemics of the clinics: beds are only separated by curtains, thus spurring spontaneous conversations among patients undergoing treatment during which information is exchanged regarding CAM as well as conventional therapies and practitioners. The clinics under scrutiny are manifestations of cultural creolization, as they are an amalgam of cultural fragments: symbols of TCM such as acupuncture diagrams; symbols of biomedicine such as blood pressure monitors; and symbols of vitalism, such as paintings depicting chakras and energy fields. Cultural creolization is evident in the conceptual and behavioral environment as well: doctor-patient consultations often involve discussions of genetic and karmic illness etiology, while energy healing is at times employed as augmenting therapy. This type of creolization is made possible by the cultural creatives populating the two clinics, the dispositions toward syncretism that the majority of both patients and staff exhibit.

The other two TCM clinics within the study exhibit an intentional modelling of the Chinese cultural environment to the extent that most symbols within the space of the clinics are linked to a Chinese identity in some way (through its language, aesthetics, origin, etc.). One TCM clinic is headed by a Chinese man, the other by a Hungarian man with formal training in China. The proxemics of the clinics and the practitioner/patient attitudes reflect less of a tendency toward syncretism, and more toward enculturation. With regards to therapeutic decision making, both types of cultural climate are in a continual dialectic with biomedical procedures and results. Whether it is considered a model to be followed or rejected, biomedicine acts as a cultural sounding board, influencing dispositions concerning the evaluation of therapeutic efficacy, expectations for the doctor-patient relationship, and so on.

## Results

Participant observation involved 105 patients (mean age = 53.9), a total of 49 first consultations were observed at the sites. In-depth interviews were conducted with 9 patients undergoing regular treatment (males *N* = 3; mean age = 58.6) and with 9 practitioners (males *N* = 4; mean age = 50.1). Data gathered with the two methods were coded uniformly (EM 1–3 and PJ 1–4; see methods section); the cumulative results are presented below.

The first milestone in the patient journey included in our study is the point of diagnosis. As biomedicine is the dominant medical system in Hungary, there is a cultural disposition toward accepting the nosology of biomedicine in defining or labeling the complaints. All patients within the scope of this research had undergone biomedical testing processes and in most cases received a diagnosis, though many arrived at the TCM clinics with either no proclaimed illness or rejecting the proclaimed diagnosis. In the former situation, a series of diagnostic tests are performed, but the results do not yield a diagnosis of a certain disease. The latter situation (patient rejecting diagnosis) occurs quite rarely, due to the Western disposition toward the biomedical categorization of illness, and happens in response to severe disease, such as cancer.

Provided the patient follows a typical trajectory of entering conventional medicine as their primary treatment option, the significance of the physician-patient relationship is heightened. Many leave western medicine (or seek complementary treatment) because they are unsatisfied with their doctor. This situation occurs when the patient enters this interaction with their own set of expectations concerning the amount of time and attention that they should receive during consultation, and these expectations frequently extend to the behavioral environment of the locale of healing as well (gestures of care, emotional support, etc.). Loss of trust within the physician-patient relationship occurs when these psycho-social expectations are not met. There are also factors in communication, which lend to loss of trust: the patient feels they did not receive an adequate explanation of their illness, the risks of the procedure, the intensity of side-effects, or the available alternative options. There are many instances also, where the patient feels the prognosis of the disease was conveyed by the doctor without empathy or left no interpretive space for retaining hope in healing. As a result of tensions in the physician-patient relationship, many patients report “*maltreatment*”, or cite being “*misdiagnosed*” or prescribed the “*wrong medicine*” in their reason for leaving conventional medicine.

Patients who end up at TCM clinics usually begin a process of biomedical treatment, but encounter side-effects they deem severe. Several patients then turn to CAM for either complementary treatments to subdue these novel somatic sensations, or lose trust in biomedicine and opt for alternative treatment. Furthermore, there are many instances of patients leaving conventional care because there is no available cure for their illness, or there is no cure available at the present stage of their disease (the illness has not yet fully developed or it is in terminal phase). Frequently, the patient will have tried one or many biomedical treatments that were not successful in alleviating a reoccurring illness or symptom. Patients with chronic disease commonly enter the world of CAM seeking not only an alternative treatment, but a novel way of interpreting their illness experience.

There are many cases where the patient is not seeking a new interpretation of illness, but seeking an interpretation that is akin to their existing concepts. In both instances, the motivation behind CAM use is so called “philosophical congruence,” which signifies a myriad of patient dispositions that are met within the cultural and behavioral environment of CAM. Choosing CAM for philosophical reasons includes issues in the domains of etiology, diagnosis, and treatment, as well as dispositions toward pharmaceuticals, vaccines, and certain types of diagnostic procedures. Philosophical congruence can appear in the realms of concepts concerning world and man, it can signify a type of religious conviction, and it can also involve cultural values connected to lifestyle or modes of exercise. There are popular dispositions among CAM users that regard keywords such as “*natural*”, “*ancient*” or “*holistic*” treatments, which engulf a wide array of beliefs and behaviors.

Finally, there are alternate routes of entry into CAM use that can occur independently from the presence of philosophical congruence or even physical illness. Many individuals (laypeople and western medical professionals alike) are “*just curious about TCM*” (or other CAM) procedures and would like to experience them firsthand, even though they are not necessarily in need of them. Others use CAM for maintaining their health, thus for preventive purposes, and do not readily employ CAM therapies in case of actual illness. Lastly, many undergoing CAM therapies are doing so because they want to treat an addiction, and may later turn to CAM for treatment of other ailments.

## Discussion

Reviewing factors and motivations behind CAM use based on the patient journey and not in the dichotomy of “push and pull,” may lend us a wider array of factors and a more in-depth understanding of the complexity at hand. The vast majority of recorded cases in the fieldwork demonstrate that biomedicine is the primary domain for the patient seeking help, yet the bedrock of each therapeutic trajectory is set by a preliminary orientation among sources of information concerning possible interpretations of complaints and therapeutic options.

## Orientation among information

Questions of healthcare are no exception to the phenomena of information proliferation; countless contributing sources ensue polycentric and diffuse information production [26], a milieu in which the individual habitually navigates amongst contradictory facts and opinions. As stated above concerning the “market” of worldviews, sources of healthcare information production (individuals, institutions, medical systems) also compete with one-another. Prior to seeking help, patients commonly immerse themselves in information they regard as relevant to their condition. The abundance of information (mainly on the Internet) necessitates a way of filtering what is “useful” and what can be disregarded; in this preliminary phase of orientation, patients already order sources of information in accordance with their previous values and preconceptions. There is a complex relationship between one’s way of filtering information (based on preconceptions) and the set of information that reveals itself, and it is not as plastic as one might think. For example, the Internet provides almost boundless information, but how one navigates it is based on preconceptions, prior associations, and what one considers to be a valid source of information.

Distinguishing between valid and false information requires points of orientation in authenticity and trustworthiness. According to Molnár, trust has withered in what were once influential societal institutions (such as the hegemony of the church, schools, parents, etc.) [24], hence points of orientation have become hazy [26]. “Truth” and “fact” are under increasingly subjective judgment – it is not the institution, but the individual that has emerged as the basis of decisions; personal experience is eclipsing tradition as the source of authenticity [26]. Firsthand experience plays a vital role in therapeutic decision making as well, as a therapy that has been utilized with success will influence its application in the future, furthermore, personal experience constitutes the basis of an information flow concerning healthcare issues among patients.

In the world of CAM, this information flow is of the utmost importance because the Other, possessing firsthand experience and/or knowledge, signifies the paramount source of authenticity. The Other may be a layperson with the same symptoms or illness, a layperson with a different illness but possessing relevant knowledge, or a naturopath with experience in treating the illness in question. Loss of trust in societal institutions thus leads to placing trust in peers and valuing information brought about by firsthand experience. Second in line to the Other’s experience is what the Other recommends, e.g.: webpage, book, institution, practitioner, therapy. Patients encountered during fieldwork regularly discuss information concerning the efficacy of therapies, products or practitioners, oftentimes crossing boundaries of illness interpretations and explanatory models.

## Biomedical diagnosis

Among most patients in the study, the initial domain of seeking help was biomedicine, which not only illustrates the primacy of western medicine as a disposition, but also sheds light on the unquestioned acceptance of biomedical nosology. If TCM patients under present scrutiny received a biomedical diagnosis, they retained it despite their choice of therapy or appropriated illness interpretations. It is worthwhile to emphasize that while a biomedical diagnosis is rarely called into question, the etiology of an illness is commonly challenged and may constitute a motivational factor in choosing CAM (see: “Philosophical congruence” below).

Failing to receive a biomedical diagnosis is a frequent reason leading patients to TCM clinics. Examples include assorted experiences of localized pain, digestive problems, musculoskeletal problems, etc. The diagnosis patients receive at the clinics are TCM-specific (syncretic or encultured); to a few patients this is insufficient, but to most, it provides enough closure for trust to develop and the treatment to begin. Consequently, if the cultural expectation of assigning an illness to a specific complaint (i.e.: diagnosis) cannot be satiated by the specificity of biomedicine, it may be fulfilled by someone bearing prestige in matters of healthcare expressing the sentiment of “knowing what is wrong” with the patient. Of course, this sentiment may only be sufficient because in such a situation biomedicine has not been able to offer this knowledge.

Thus, there is a cultural disposition of receiving a diagnosis whereas the complaints become symptoms and construe a treatable illness. Most individuals turn to biomedicine as the authority for giving a name to their somatic ailment, and provided the diagnosis is acceptable for the patient [31], they retain the western diagnosis. In cases where patients do not receive a biomedical diagnosis, they turn to CAM for this indispensable easement. If the somatic ailment would remain without a name, it would create the state of Geertzian chaos [32] and the individual would have immense difficulty coping with the situation.

## Biomedical consultations

In a typical trajectory, the patient enters biomedicine seeking a diagnosis while coming into contact with one or several medical doctors; it is at this point that the doctor-patient relationship becomes a relevant factor in therapy choice. Over the course of the fieldwork, patients verbalized several needs that contributed to their choosing TCM as a complementary or alternative therapy. These needs pertain to the biomedical practitioner, as well as to the behavioral and physical environment of healing.

Concerning the doctor-patient relationship three main needs are vocalized with the biomedical model acting as a reference point: the need for a lengthier consultation, the need for qualitative attention from the doctor, and the need for being unrestrained in one’s narrative of distress. These can be considered lesser needs lying in the backdrop of the motivations of therapy choice because they are usually not the reasons patients attribute to choosing TCM.

The need for being unrestrained in one’s narrative of distress proves to be a key expectation because, as evident in the praxis of most medical doctors, the patient arrives to the first consultation with a set of preconceptions concerning their illness, assumptions constructed via their mode of filtering information. Consequently, on the one hand, the patient wants to impart their interpretations regarding their illness, frequently even psychosocial factors they accredit to their physical problem, and on the other hand, they expect a manner of communication from the doctor, which they understand. “Understandable communication” is not only a matter of semantics, but also of semiotics: most patients prefer to interpret their somatic ailment in a social and psychological context [31, 33, 34], which is made possible in the medical system of TCM extending into the affective domain as well. TCM consultations are chiefly led by the patient; this implies that the patient is free to steer the conversation to any semiotic domain they see as relevant to their condition. Empathetic attention in this particular instance means the doctor considers the factors elaborated by the patient as pertaining to the medical explanatory model they are co-constructing and, in a rhetorical and semiotic sense, does not unilaterally impose their own explanatory model on the patient.

Aside from length of consultation, quality of attention, and aspects of communication, which all pertain to the practitioner-patient relationship, there are two more vocalized needs that involve the behavioral environment of the locale of healing. Patients of TCM clinics often vocalize their need for and satisfaction with the staff’s gestures expressing care. Finally, a need constituting the backdrop for therapy choice is one concerning the proxemics and the behavioral environment: patients regularly remark that they “*do not feel sick*” when they are at these clinics. Based on patient accounts, the ethos of such a clinic tacitly inspires the construction of a social network and a reaffirmation of social support, which many patients lack in their life outside of the clinic.

Another aspect of therapy choice connected to biomedical consultations is the patient losing trust toward a physician or toward biomedicine in general, which can occur due to several reasons. Initially, the patient may feel they have received an insufficient explanation for their condition, such as the case of a young woman diagnosed with an ovarian cyst and upon her inquiry about the illness she was “*merely*” given the explanation that it had developed “*due to hormones*.” She found the explanation inadequate resulting in her turning away from western medicine. Sometimes the biomedical explanation will be sufficiently detailed for the patient, but the patient does not agree with the etiology or explanation of the illness. Loss of trust may also occur if the patient feels they were not adequately informed concerning the risks or side-effects of an impending treatment/procedure, or concerning other therapeutic options they could take. Also, frequently patients recount they “*found out*” they had been “*misdiagnosed*” or prescribed the “*wrong medicine*” by their biomedical doctor or report other instances they ascribe to “*maltreatment*.” It may be posited that these sentiments are indications of the loss of trust in the physician-patient relationship, rather than its causing agents.

Loss of trust can also occur due to contradicting physician opinions (in diagnosis or treatment), as the patients feels “*neither* [*doctor*] *knows what they’re talking about*”. A patient may also lose trust if they receive a prognosis that they do not agree with or it is not communicated by the physician in a manner that is acceptable for the patient. An example of the latter is taken from a 55-year-old woman’s narrative who was grappling with breast cancer. This patient reported her physician telling her she does not have long to live by stating, “*Your time is up*.” The woman decided to discontinue her cooperation with that doctor and utilize TCM as a complementary therapy; she divulged that although the content of the prognosis was difficult to hear, it was the manner in which it was communicated, setting off emotions of anger and distrust, which led her to switch physicians and seek an augmenting therapy.

## Biomedical treatment

There are times when diagnosed patients are confronted with the fact that western medicine cannot offer a cure for their illness and thus turn to CAM, many of whom are chronically ill and are looking for avenues of symptom management (cf: [35]). At other times, biomedicine may not be able to offer a cure at the present stage of a certain illness. Thus hearing physician remarks such as “*come back when the problem becomes permanent*” and “*until then, all we can do is wait*” induces a feeling of helplessness in the patient that will drive them to seek out other forms of medicine. A similar situation develops if it is too late for a biomedical cure, that is, the disease is in its terminal phase and solely subject to palliative care.

When asked about their reason for turning to TCM, patients often cite that they had tried the western medical cure, but it was ineffective. This is most common among patients with chronic or reoccurring illnesses and signifies a well-documented motivational factor in CAM use [11, 36]. Another central reason behind complementary medicine usage is alleviating the side-effects of a biomedical treatment or procedure, yet it is very common to see patients autonomously discontinuing a biomedical treatment plan because the side-effects are deemed “*unbearable*.” At 2 out of the 4 clinics, there are instances of patients with Parkinson’s discontinuing medications prescribed by their physicians due to the intensity of side-effects, and seeking various alternative medical treatments and products to ease their primary symptoms.

There have been a variety of studies conducted on whether patients inform their biomedical doctors concerning employed complementary or alternative treatments. Many authors found that patients generally do not disclose CAM use to their physician [36, 37], but the opposite has also been documented, where all participants of the study readily informed their doctor about the topic [35]. Based on several studies, Faith reports that “reasons for lack of disclosure include concerns about negative reactions or judgment from providers, perceptions that CAM use is not something about which providers need to know, and providers not initiating discussions about CAM” [36]. These findings directly correlate with the results of the current study as well. Whether the patient informs their doctor about their decisions concerning CAM is dependent on many factors, such as level of trust, mode of communication, type of CAM employed and its social appraisal, as well as the interaction of CAM and biomedicine in a micro and macro socio-cultural context.

## Philosophical congruence

The last motivational factor in utilizing CAM is the complex skein of “philosophical congruence” [38], that is, cultural elements of a CAM modality are congenial to some aspect of a patient’s explanatory model or personal values. CAM is associated with notions of holism, mind-body unity, wellness, vitalism (energy-concepts), and cooperative healing [15]. According to Stratton (cf: [39]), the more an individual espouses these values, the more likely they are to turn to CAM; in the late 90’s, Astin found that 15–17% of Americans utilizing CAM did so due to “philosophical reasons and a preference for dealing with ailment alone” [38]. This philosophical congruence may involve concepts of world, man, illness (etiology and treatment) and (a “holistic” understanding of) health, but this is not an easily circumscribable, unified explanatory model. Correspondingly, CAM is an aggregate name for very different types of medical systems, thus what is “philosophically congruent” is varied, and difficult to define from both directions. Perhaps it is more apt to speak of explanatory model “elements” that individuals identify with in a process of information-seeking and cultural creolization.

The most powerful explanatory model elements, in terms of congruence and motivation to choose CAM, concern the etiology of illness. As Kleinman [33] and many others have posited, individuals prefer thinking of illness in psychosocial terms, which can also be regarded as a “pull factor” toward CAM. Yet, upon closer scrutiny, this is a vastly documented, complex human need and is hence not necessarily in direct opposition to western medicine’s paradigm. Identifying a pathogen or a genetic cause may often leave the illness without meaning for the patient, and construing meaning for an illness seems especially vital in the case of chronic, reoccurring and terminal diseases or when there is no biomedical cure available.

Thus, subscribing to a psychosocial etiology is widespread among patients, often transgressing discrete illness boundaries and coalescing with concepts of world and man. Tracing illness back to emotional or cognitive factors implies the individual has control over their recovery as well, provided the psychosocial tensions are resolved. As opposed to biomedical culture, CAM presents a rhetoric and symbology that is easily understood by the patient and can be converted to personal meaning. Accordingly, one word uttered in a specific cultural context may be imbued with more meaning than a complex medical explanation for an illness. For example, in the case of a young man, a TCM practitioner mentioning he had a “*weakness of the lung*” lead him to remark in concurrence that he had been “*feeling down lately*,” thus yielding a discrete psychosocial etiology to his physical problem. This case could lead into the arena of illness symbolism, a strong factor in philosophical congruence, but will not be discussed here because it warrants a separate analysis.

In terms of philosophical congruence, CAM has been linked to Modern Health Worries (MHWs) [40, 41] as well, thus etiologies such as electrical pollution, food additives and other chemicals often lead individuals to CAM use. These etiologies are in a close dialectic with societal values, signifying an opposition to consumer society, urbanization, fast-paced lifestyle, and so on. Spawned partly from this disposition is the preference for the “*natural*,” which is a driving force in choice of therapy. Congruently, many patients also retain an anti-drug disposition, refraining from the use of pharmaceuticals when possible, or even adhering to an extreme and refusing any kind of said treatment. Several patients and CAM practitioners vocalize a belief that the pharmaceutical industry has an interest in “*keeping sick people sick*,” hence the preference for the “*natural*” is on par with an intense loss of trust in many aspects of the healthcare industry.

Adjacent to the above dispositions lie convictions concerning treatment, which are embedded in worldview and retain a reciprocal relationship with concepts of etiology, illness and health. At the clinics there are instances of cancer patients who, for example had their tumor removed, but decided to discontinue the biomedical process and refuse the subsequent chemotherapy and/or radiation therapy. This view regarding therapy may be connected to a disposition toward “*natural cures*,” but may also stem from notions of etiology. If one gives credence to the conviction that “*cancer is not an illness, but a sign of a weak immune system*,” then it is consistent to assume that chemotherapy would “*destroy the weakened immune system*.” Some patients only suspect they have a tumor, but are not certain, because they had rejected having a biopsy based on the premise that a tumor is the body’s way of “*sealing off*” a damaged area and “*tearing it open*” with a biopsy would lead to metastasis. Insofar as an individual valuates any kind of biomedical procedure or therapy as “*life-threatening*,” they are likely to turn to CAM, even without underlying philosophical congruence.

Another element of philosophical congruence in dialectic with societal values is a preference for the “*ancient*.” Practitioners at the clinic and many patients tend to be open to syncretizing not only Eastern medical systems, but also Western traditions into a conglomerate of acclaimed “*ancient knowledge*.” Concepts of “*ancient Hungarian*” culture and shamanism are habitually regarded as belonging to a pool of “*ancient wisdom*” practiced by all in a mythical time and linked to the various traditional medicines on a global scale. This disposition may be regarded as a “cultural creative” reaction to cultural fragmentalization and a coping mechanism for globalization. Paradoxically, this preference may also signify an opposition to globalization, exhibiting a nationalist edge, e.g.: espousing the belief that TCM has its roots derived from Hungarian forbearers migrating to present day Hungary from the periphery of the Chinese Empire.

A preference for the “*natural*” and the “*ancient*” often entail a preference for “*holism*,” although many times holistic attitudes occur independently of these dispositions. Among patients of the clinic, holism is generally understood in two ways; its first version entails a reaction to dualism and concerns viewing man as a whole, a fusion of culturally reified constituents: body, mind, soul, spirit, etc. Most CAM modalities are congruent with this notion of holism; TCM considers the body in terms of systems (in accordance with meridians), but incorporates the affective domain as well. The second notion of holism espoused by clinic patients is a reaction to reductionism and increasing specialization within the conventional medical system. Patients experiencing several complaints or illnesses long to consult one physician concerning all of these, rather than adhering to the compartmentalization rampant in western medicine.

Philosophical congruence may also come into play concerning dietary or exercise preferences constituting an “entrée” [42] into the world of CAM. Dietary preferences often correlate with loss of trust in societal institutions, as they are underlined by assumptions concerning the chemical treatment of food, the proliferation of preservatives, and other notions regarding the mass production of sustenance. Exercise preferences, such as yoga and martial arts, can lead to CAM use as well, insofar as the mode of exercise is embedded in a(n Eastern) philosophical system and the individual identifies with these cultural values. It is noteworthy that individuals may also receive guidance concerning diet and exercise from a biomedical professional, but oftentimes may give more credence to sources of authority in CAM, perhaps due to a lack of an underlying philosophical system as a motivational force.

## Alternate “entrées”

Employing a complementary or alternative therapy may occur outside of the patient journey as well, not connected with a specific illness. Such entrées include curiosity, prevention, and addiction. In the case of some “patients,” their reason for being there is that they are “*just curious*” about what an acupuncture treatment feels like or that they are interested in health promotion. Yarney states that 31.2% of CAM users belong to this category and asserts that this signifies a doubt concerning the effectiveness of conventional treatment [3]. This is not necessarily true, as pure curiosity may occur due to the “*exotic*” nature of acupuncture in a Hungarian (or Western) setting, and its employment is restricted to “*trying it out*.” Curiosity is a factor among healthcare professionals as well, there were a few medical doctors recorded during the fieldwork who wanted to understand the empirical foundations of acupuncture and described themselves as “*open*” to new experiences. Finally, the treatment of addiction (mainly alcohol and nicotine) is not rare in TCM, as this seems to be a territory within Hungarian healthcare where medical doctors are more comfortable referring patients to CAM, and patients more readily associate CAM modalities as competent in aiding substance withdrawal. A successful treatment in substance withdrawal may signify an entrée into the world of CAM and lead to regular CAM use for other illnesses as well.

## Conclusions

The article has endeavored to compile factors in choosing complementary or alternative medicine, more specifically, Traditional Chinese Medicine as a treatment for one’s illness. In effort to avoid the dichotomy of “push and pull factors,” the panoply was structured around milestones of the patient journey. The qualitative research design itself and the structuring of results aimed to present information concerning therapy choice with full consciousness of the difficulties in defining CAM and quantitatively measuring CAM use. Studies and thus statistics vary according to research methods, socio-cultural context, and CAM categorization. Prolonged participant observation and scrutiny from the perspective of the patient journey offered insight into the intricacies of therapy choice and its socio-culturally embedded reality.

The influence of one’s explanatory model (consisting of concepts of world, man, illness, and health) is ubiquitous throughout the therapeutic trajectory from the onset of the patient journey. Preconceptions and previous experiences are a vital force in the first milestone of the patient journey, that is, orientation among sources of information or more specifically, the process of searching for and appraising health-related information. Subjective experience (firsthand, or that of the Other) signifies the most reliable source of information in matters of illness. This statement extends to recommendations concerning CAM modalities, CAM practitioners, and even to biomedical physicians. The theme of (building and loss of) trust is present throughout the patient journey, a determining factor in patient decisions and dispositions toward both CAM and biomedicine.
